# An integrative network analysis framework for identifying molecular functions in complex disorders examining major depressive disorder as a test case

**DOI:** 10.1038/s41598-021-89040-7

**Published:** 2021-05-06

**Authors:** Anup Mammen Oommen, Stephen Cunningham, Páraic S. O’Súilleabháin, Brian M. Hughes, Lokesh Joshi

**Affiliations:** 1 Advanced Glycoscience Research Cluster (AGRC), National University of Ireland Galway, Galway, Ireland; 2 Department of Psychology, University of Limerick, Limerick, Ireland; 3 School of Psychology, National University of Ireland Galway, Galway, Ireland; 4 Centre for Research in Medical Devices (CÚRAM), National University of Ireland Galway, Galway, Ireland; 5 Health Research Institute, University of Limerick, Limerick, Ireland

**Keywords:** Computational biology and bioinformatics, Neuroscience, Biomarkers, Diseases

## Abstract

In addition to the psychological depressive phenotype, major depressive disorder (MDD) patients are also associated with underlying immune dysregulation that correlates with metabolic syndrome prevalent in depressive patients. A robust integrative analysis of biological pathways underlying the dysregulated neural connectivity and systemic inflammatory response will provide implications in the development of effective strategies for the diagnosis, management and the alleviation of associated comorbidities. In the current study, focusing on MDD, we explored an integrative network analysis methodology to analyze transcriptomic data combined with the meta-analysis of biomarker data available throughout public databases and published scientific peer-reviewed articles. Detailed gene set enrichment analysis and complex protein–protein, gene regulatory and biochemical pathway analysis has been undertaken to identify the functional significance and potential biomarker utility of differentially regulated genes, proteins and metabolite markers. This integrative analysis method provides insights into the molecular mechanisms along with key glycosylation dysregulation underlying altered neutrophil-platelet activation and dysregulated neuronal survival maintenance and synaptic functioning. Highlighting the significant gap that exists in the current literature, the network analysis framework proposed reduces the impact of data gaps and permits the identification of key molecular signatures underlying complex disorders with multiple etiologies such as within MDD and presents multiple treatment options to address their molecular dysfunction.

## Introduction

Depression and other common mental disorders constitute half of the leading causes of disability worldwide^1^. It has recently been suggested that mental illness accounts for 32.4% of years lived with disability and 13.0% of disability-adjusted life-years and an estimated cost in excess of £2 billion and US$ 40 billion every year for the employees in United Kingdom and United States respectively^2,3^. Among different forms of mental disorders it is estimated that depression disorders (a mood disorder included in Diagnostic and Statistical Manual of Mental Disorders: DSM), is prevalent in ~ 4.4% of world population^1^. Studies examining long-term mortality trajectories consider major depressive disorder (MDD), a major form of depressive disorder, as a critical mortality risk factor^4^. Epidemiological studies have demonstrated that the co-occurrence of diverse components of metabolic syndrome (high blood pressure, lipid abnormalities, hyperglycaemia and central obesity) with MDD, is associated with a fourfold increased risk of cardiovascular disease^5–7^. Diverse factors attributed in the development of MDD include somatic illness, early life stress^8^, socioeconomic factors^9^, and genetic factors^10^. Depending on the subtype of MDD (e.g., melancholic, psychotic, catatonic, and atypical depression)^11^ as well as diverse contributing factors, depressive symptoms are typically manifested as physical and emotional exhaustion, lack of interest or anhedonia, insomnia, impaired concentration, cynicism (depersonalization), and culminate in psychophysiological endpoints such as suicidal ideation and behaviour^12,13^.

An appreciation of the complex relationship between the clinical symptoms of MDD and its underlying causal biomolecular network is crucial for disease diagnosis as well as clinical management of the disease. Network biology based approaches, coalescing diverse OMICs data with biomolecular interaction networks, have emerged as a powerful integrative and systems-level approach in understanding complex disease-disease and disease-biomolecular associations^14–16^. When applied, such an analytic approach involves a dimensionality reduction of altered state of biomolecules to more comparable and interpretable set of biological pathways or molecular network information, providing significant insight into the biological mechanisms underlying a disease pathology^17–20^. Such integrative network analysis approaches have significantly aided in identifying putative diagnostic biomarkers, therapeutic targets and pathophysiological mechanisms underlying complex diseases^21–23^. Furthermore, the continued generation of large and publicly-available ‘omic’ data resources makes real the opportunity to examine complex disorders through a tiered systems biology approach, combining data collected across independent groups of individuals to identify novel biological parameters and potential causations, aiding to greater clinical interpretation and management. Complex neuropsychiatric diseases such as MDD, attributed by etiologic heterogeneity and a major comorbid risk factor for metabolic syndrome^24^, can be examined and elucidated at a greater molecular level by such an integrative systems biology approach^25^.

A number of studies had proposed comprehensive analysis framework at the systems level for characterizing functional biological pathways of candidate genes, proteins and metabolites in MDD^26–31^. The findings and of these studies, focusing on identifying diagnostic markers and pathophysiological mechanisms associated with the symptoms of MDD revealed a bidirectional interaction between systemic stress, chronic low grade inflammatory response, altered neuronal pathways and synaptic signaling in the pathogenesis^28–31^. Accumulating evidence suggest that the etiopathogenesis of MDD largely involve biochemical abnormalities in monoaminergic systems (serotonin, norepinephrine, and dopamine), glutamatergic neurotransmission, and structural abnormalities in the prefrontal projection systems^32–34^. The majority of these pathway-centric enrichment analysis methods has largely been dependent on comprehensive sets of online databases, which differ significantly in terms of representation of biological pathways and statistical enrichment analysis conducted^35^. Additionally, these databases often under represents or lack the relevance of post-translational modifications (PTM), a critical regulator of core biological pathways under health and disease conditions. For example, emerging data indicate alteration in glycosylation PTM, a critical modulator of neuronal functions and immune responses^36–38^, to be significantly correlated with the inflammatory phenotype observed in MDD subjects. As examples of altered glycosylation, levels of the sialylated glycan structure Neu5Acα2-6GalNAc on plasma proteins^39^, high branching plasma *N-*glycans with tri and tetra-sialylation in males and monogalactosylated *N*-glycome on IgG4 subclass in females^40^, non-fucosylated biantennary glycans as well as α1-3-fucosylated triantennary glycans have all been shown to be significantly correlated with inflammatory cytokines and pathways in MDD subjects^41^. Moreover, majority of the systems biology approaches focusing primarily on transcriptomic or genomic data driven pathway enrichment analysis, often lack integration of non-genetic molecular measurements generated from the clinical samples. These data resources often available in the form of evidence based umbrella reviews; meta-analysis or systematic reviews provide a reliable source of molecular markers that are significantly associated with the disease phenotype.

In the current study, an integrative network analysis framework for the detailed biological process enrichment analysis to compile a comprehensive coverage of core biological processes associated with the MDD phenotype was undertaken. Diverse publicly available transcriptomic datasets associated with the MDD phenotype along with reported protein and metabolite markers, where subjected to detailed biological process enrichment analysis. In parallel and to compliment these datasets the biological role and significance of glycosylation PTM (lectins as well as glycoconjugate structures) was undertaken and integrated into the network analysis to assess correlation with the neurological function and inflammatory responses which are reported cellular dysfunctions in MDD subjects^32–34,42–50^.

## Methods

**Figure 1:**
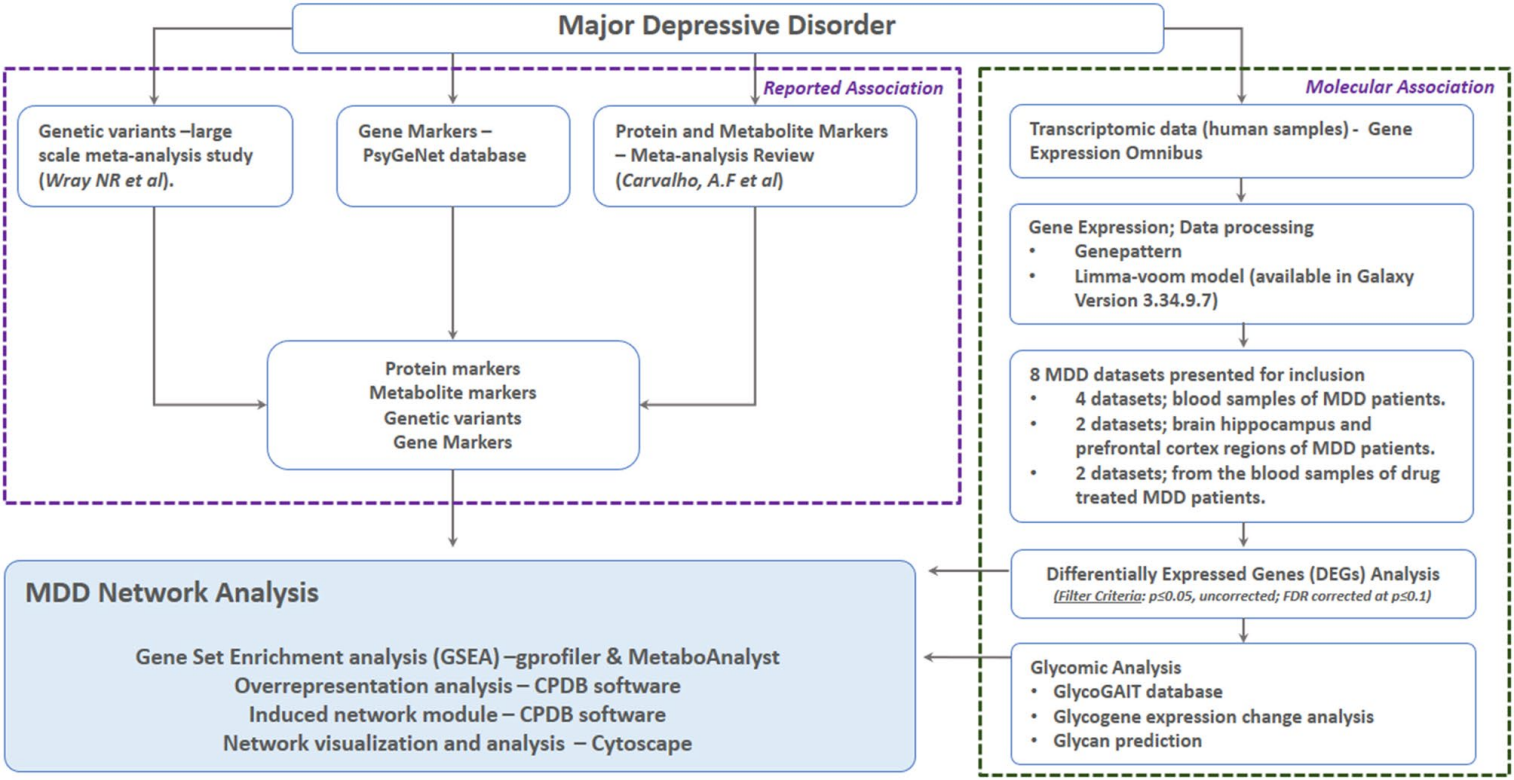
A flowchart depicting the sequential steps adopted in the current study for the integrative data analysis methodology. Various online software analysis tools as mentioned in the diagram were leveraged along with our proprietary GlycoGAIT database for detailed gene set enrichment analysis, complex protein–protein, gene regulatory and biochemical interaction pathway analysis.

The design methodology explored for the integrative analysis encompassing transcriptomic assessment, reported bibliographic databases, open knowledge platforms and ‘omic’ databases is depicted in Fig. 1. The goal was to evaluate the functional significance and potential of this methodology in the identification of molecular function and biological pathway association of genes, proteins and metabolites for MDD, extending to the examination of the role of glycosylation PTM.

### Gene expression data selection, bibliographic search and knowledge databases

For the transcriptomic analysis, public database Gene Expression Omnibus (GEO) (https://www.ncbi.nlm.nih.gov/geo/) was queried using the key word “Major Depressive Disorder”. Datasets included both blood and brain samples from MDD subjects and only those datasets with ≥ 3 replicates for both test and control samples were considered. Datasets without gene annotation were excluded from the analysis. For understanding the gene expression changes under treated conditions, data obtained from MDD subjects treated with anti-depressants drugs (ADD) were also included (using the generic names of ADD, Suppl. Table 1). As the number of significant datasets was low, data generated in cell line and in vivo model system were incorporated. For data processing and gene annotation, online software tools GenePattern (http://software.broadinstitute.org/cancer/software/genepattern/)^51^ and Galaxy (https://usegalaxy.org/)^52^ were used throughout, following the procedures detailed in user manuals. In GenePattern, preprocessing of RNA-Seq data including normalization, missing value imputation, collapsing multiple probe set expression values into a single expression values were performed using the VoomNormalize (v2); ImputeMissingValues and CollapseDataset modules respectively. Differential expression of the preprocessed data files were then performed for individual datasets using the ComparativeMarkerSelection module^53^. While, in Galaxy the limma-voom tool was utilized for the differential expression analysis accepting the default Trimmed Mean of M values (TMM) normalization method and applying filters to remove lowly expressed gene in each datasets selected for the analysis (we choose to retain genes if they are expressed at a counts per million above 0.5 in at least three samples). Significance of marker genes were calculated using default *p*-value adjustment methods in analysis^54^. For few datasets normalized, log2 transformed gene expression matrix files were used directly from the GEO databases, wherever available.

A systematic query of the PUBMED bibliographic database^55^ using the search query [“major depressive disorder” (Title/Abstract)] AND [biomarker(Title/Abstract)] was performed. After filtering results using “review paper” and “full text” as parameters, a comprehensive umbrella review by Carvalho et al.^56^ was identified reporting evidence based correlation of protein and metabolite markers for the 5 most prevalent and high burden major mental disorders. An Umbrella review systematically evaluates and collects information from multiple systematic reviews and meta-analyses on all outcomes of a given topic for which these have been performed, eliminating or greatly reducing reporting bias^57^. From Carvalho et al., protein and metabolite markers reported with ‘strong correlation’ with the MDD phenotype was presented for integrative analysis.

Through PsyGeNET (Psychiatric disorders Gene association NETwork), genetic markers reported to be associated with MDD phenotype were compiled^58^. Genetic variants from a published meta-analysis of 130,644 MDD cases^59^ was also examined for detailed functional genomic data enrichment analysis to build a comprehensive pathway coverage of reported genetic and non-genetic markers associated with MDD.

### Glycosylation process related gene pool

For the gene expression analysis of glycosylation process related genes, the GlycoGAIT database^60^, an interactive web database developed within our research group was utilised. For this analysis, the GlycoGAIT database was enriched through manual capturing of enzymatic reactions for the glycosyltransferase and glycosidase enzymes, which were extracted from the BRENDA enzyme database (https://www.brenda-enzymes.org/index.php) and ExPASy bioinformatics resource portal (https://www.expasy.org/). For a number of interactions, where the reaction information is not available, interactions were curated manually from PUBMED sources. The reaction formats as well as the substrate and ligand short form representation are manually formatted to make it consistent throughout. Information on proteoglycan genes were extracted from the HGNC database (https://www.genenames.org/). For linking glycosylation reactions to glycan structure predictions, well characterized human glycan epitope structures were also captured by manually selecting epitope structures from the GlycoEpitope database (https://www.glycoepitope.jp/epitopes/epitope_list). This list was further enriched with additional epitope structures reported to be present on immune cells from published literature sources^61–64^.

### Nomenclature mapping and gene family association

Prior to enrichment analysis, mapping of entity names to their respective standard nomenclatures were performed for metabolite entities using Chemical Translational Service (http://cts.fiehnlab.ucdavis.edu/) and for the protein and gene entities using the multi-symbol checker tool in HGNC database. This standard nomenclature mapping ensures no metabolite, protein or gene markers are missed during the enrichment analysis due to name mismatching. For detailed enrichment analysis, the dimensionality of the differentially expressed genes (DEGs) were reduced by generating smaller subset of more closely associated genes through mapping individual genes to respective gene families using the BioMart software available through HGNC web interface (https://biomart.genenames.org/). Top ranking gene family clusters were then identified from this list using the built-in statistical function in Excel “RANK function”.

### Gene set enrichment analysis, cellular process mapping and network visualization

Functional enrichment analysis of the literature based protein markers was performed using the open-source software and web server—gProfiler^65^. The HGNC gene symbol of the proteins were used as the query in the g:GOST functional profiling interface in gProfiler using Homo sapiens as the organism species, g:SCS threshold as the significance threshold and the user-defined *p*-value threshold as 0.05 in the advanced options (Suppl. Table 2a). Similarly, for the literature based metabolite markers enrichment analysis was performed using the web-based analytical pipeline for high-throughput metabolomics data analysis—MetaboAnalyst^66^ and the ConsensusPathDB—(CPDB—http://cpdb.molgen.mpg.de/)^67^. For the enrichment analysis, metabolite name mapping was performed manually to their respective KEGG, PUBCHEM and HMDB IDs^68–70^. Due to greater coverage we used CPDB for subsequent analysis by inputting the KEGG IDs using the overrepresentation analysis for metabolite set function. The default list of 13 pathway-based databases were selected with a *p*-value cutoff 0.01.

For gene set enrichment analysis (GSEA), HGNC gene symbols of the DEGs were used as the query input in the enrichment analysis tools such as the ‘analysis’ feature available in the Enrichr tool^71^ or the ‘over-representation analysis’ feature available in CPDB^67^. Besides, the induced network module analysis feature in CPDB was also used for identifying possible functional relationship between DEGs such as protein–protein, gene-protein and protein-metabolite reactions. The interaction network model generated for the DEGs is extracted as an XML-based file format using the CPDB visualization interface and ran through Cytoscape software (http://www.cytoscape.org/)^72^ for network visualization and analysis. The interaction edges are colored based on the nature of interaction and size of each node in the network is adjusted based on “neighborhood connectivity” wherein small size represents less connected entities and large size represents highly connected entities. Colours of the nodes are adjusted based on gene expression data.

## Results

### Functional enrichment analysis of markers identified from literature sources and knowledge databases

From the meta-analyses review published by Carvalho, et al.^56^, 7 protein markers and 10 metabolite markers were identified that were significantly associated with MDD subjects. Functional enrichment analysis of these protein markers revealed cell proliferation, cell differentiation and growth factor signaling pathway as the major categories of biological pathways represented by the protein markers. Similarly, for the metabolite markers result obtained from the CPDB and MetaboAnalyst platform was primarily enriched in pathways associated with amino acid, nicotinamide, arachidonic acid and antioxidant metabolism (Suppl. Tables 2a, 2b, 2c; Suppl. Fig. 1). Biological processes identified from the enrichment analysis of the 62 genetic markers from the PsyGeNet database were broadly related to synaptic signaling, neurotransmitter metabolic process, neurotransmitter transport and neuron development (Suppl. Table 3a,b). Additional enrichment analysis of genetic variants identified from a recently published meta-analysis of 130,644 MDD cases^59^ was performed. 67 gene were identified from this study, which either contain or are in linkage disequilibrium with a reported single nucleotide polymorphism. The GO biological process association of these gene markers were significantly enriched with the neuron generation and development processes (Suppl. Table 4a,b).

### Differentially expressed genes

Using Comparative Marker Selection model (Genepattern software) for the microarray data and Limma-voom model (Galaxy Version 3.34.9.7) for the high throughput sequencing data^73,74^, 8 out of 22 datasets were identified (Suppl. Table 5) that contained significantly differentially expressed genes (DEGs) based on the filter criteria set out (*p* ≤ 0.05 uncorrected, FDR corrected at *p* ≤ 0.1). The selected datasets from MDD subjects includes 4 datasets generated from the blood samples and 2 datasets from the brain hippocampus and prefrontal cortex regions. To compare the gene expression pattern under pathological and treated conditions, transcriptomics data under ADD treated conditions were also included in the analysis. However, owing to the current lack of datasets from the brain samples, only two datasets from the blood samples of ADD treated MDD subjects were included within the analysis undertaken. The DEGs identified after the filtering were further narrowed down by applying a cut-off value of ~ twofold up or down regulated genes. The resultant DEGs were pooled into three categories (Suppl. Table 6), based on the sample sources and types, which is as, detailed below:

(i) Blood samples, untreated—228 upregulated genes and 198 down regulated genes.
(ii) Brain samples, untreated—608 upregulated genes and 738 downregulated genes.
(iii) Drug treated, blood samples—215 upregulated genes and 129 downregulated genes.

### Gene family association and GSEA of DEGs

DEGs from the blood and brain samples of MDD subjects and from the blood samples of ADD treated MDD subjects were mapped to their respective gene families (Suppl. Table 7a). Among these, the top ranking gene families were identified by applying a cut-off value of ≥ 5. This method enabled to systematically narrow down the significant DEGs which covered ~ 14% to 22% of the genes across three sample categories which include (i) 46 upregulated and 42 downregulated genes from the blood samples (ii) 98 upregulated genes 115 downregulated genes from the brain samples (iii) 47 upregulated genes 27 downregulated genes from the drug treated blood samples. A limited number of significant glycogenes were identified among the DEGs across the three data categories, with maximum representation from the brain samples (Suppl. Table 7b).

### Glycosylation process related gene pool

GlycoGAIT database, compiled together from multiple online databases, captures 564 proteins which represents the unique list of well characterized unique list of lectins, enzymes, transporters and other proteins involved in glycosylation PTM along with a list of 38 genes categorized as proteoglycans (Suppl. Tables 8–10), collectively referred to as glycogenes hereafter. Information on 331 glycosylation reactions that represent 53 glycosylation reaction patterns in the network format (Suppl. Tables 11, 12) are also incorporated.. In addition to glycosylation reactions, the GlycoGAIT database also captured 194 well-characterized glycan epitope structures (Suppl. Table 13).

### Enrichment analysis of DEGs—blood samples of MDD subjects

GSEA and the GO biological process overlap analysis of the top ranking clustered gene families (cut off value ≤ 5) for the blood sample DEGs primarily showed enrichment of immune signaling pathways such as cellular response to cytokine stimulus, positive regulation of immune response, platelet activation, neutrophil mediated immunity, markers of myeloid—lymphoid maturation, complement—coagulation cascades and glycosaminoglycan metabolism. Key cellular processes identified for the glycogene subset involve mucin type *O*-Glycan biosynthesis and chondroitin sulfate/dermatan sulfate metabolism (Suppl. Table 14). Significant gaps in process association was observed in various pathway databases for the glycogene subsets.

**Figure 2:**
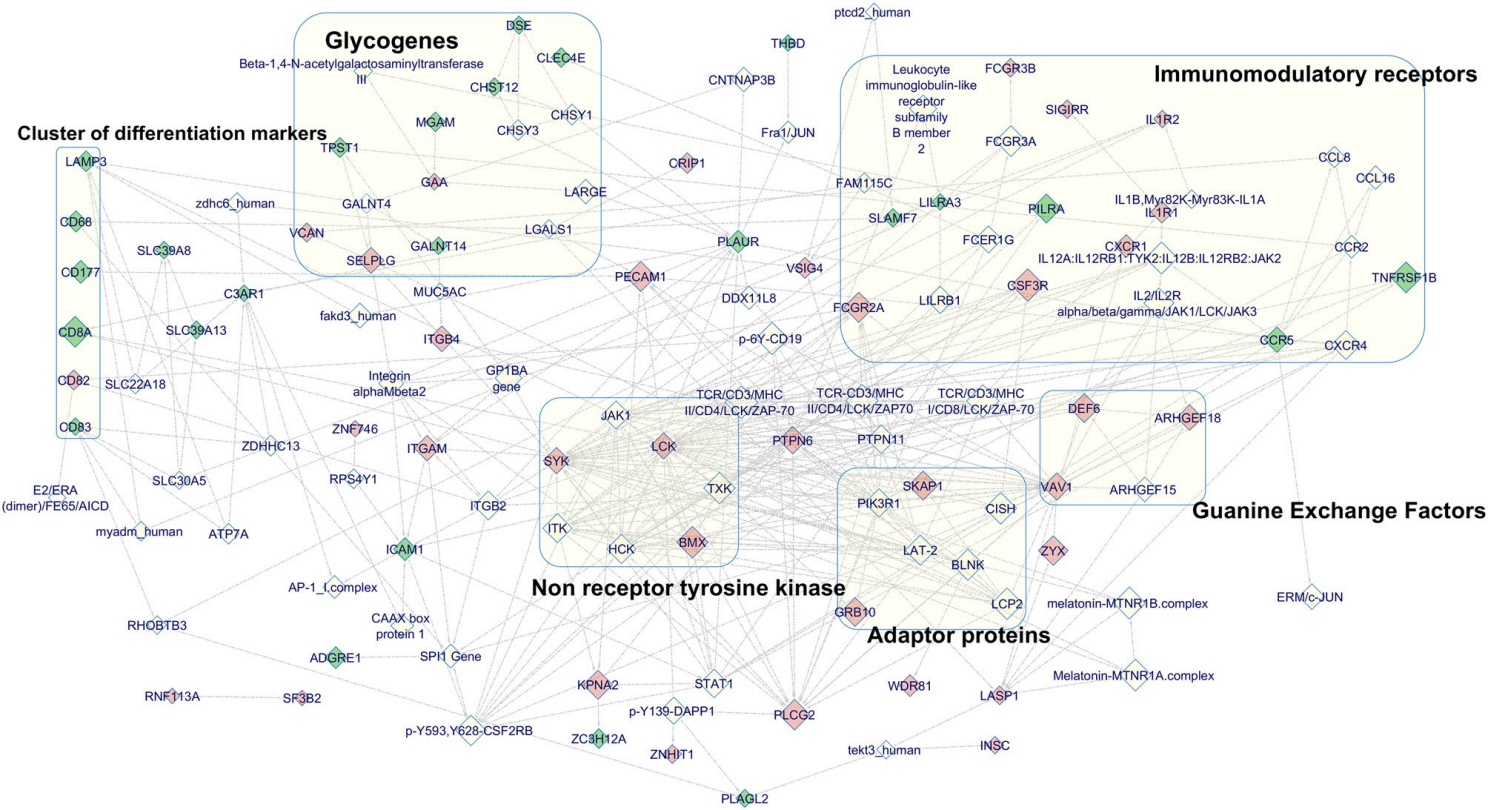
Network visualization of the integrated network module of both upregulated and downregulated genes identified from the blood samples of MDD patients. The interaction network is generated using the CPDB induced network modules by inputting the gene subsets from the top ranking gene family clusters along with the glycogenes. The compact subnetwork is created by applying a z-score threshold of 20 in the CPDB user interface. Exported network model from the CPDB is processed in the Cytoscape visualization tool using GeneMANIA Force Directed Layout and the entities are manually aligned. Colours of the nodes are adjusted based on gene expression data wherein wine red colour represents upregulated genes and dark green represents the downregulated genes. Major network modules with maximum representation of the DEGs are highlighted in the graph using a light yellow background.

Molecular mechanisms underpinning the GSEA results and the over-representation analysis results were studied by using the induced network module available in CPDB^67^. The interconnected network revealed a strong inflammatory signaling pathway comprising of immunomodulatory receptors, guanine exchange factors, adaptor proteins and non-receptor tyrosine kinases (Fig. 2). Downregulated genes, identified to be linked with this inflammatory signaling cascade, comprised of primarily markers of myeloid and lymphoid maturation that belongs to cluster of differentiation family^75–79^; few anti-inflammatory proteins such as PILRA (paired immunoglobin like type 2 receptor alpha)^80^, SLAMF7 (SLAM family member-7)^81^ and LILRA3 (leukocyte immunoglobulin like receptor-A3)^82^. Other category identified from this group consist of proteins involved in cell adhesion and migration such as PLAUR (plasminogen activator, urokinase receptor)^83^, CLEC4E (C-type lectin domain family 4 member E)^84^ and ICAM1 (intercellular adhesion molecule-1)^85^.

Protein–protein interaction network captured the association of glycogene DEGs majorly with the anti-coagulant protein PLAUR^86^ and the Integrin signaling cascade (Fig. 2). Binary interaction network between the upregulated glycogene GALNT14 and the proteoglycan VCAN (versican) with the SELPLG (selectin P ligand)—a critical protein involved in leukocyte trafficking^87^, might indicate enhanced signaling pathways for leukocyte adhesion. Tyrosine sulfation of leukocyte adhesion molecules, chemokines and chemokine receptors has been implicated in promoting atherosclerosis by enhanced recruitment of mononuclear cells^88,89^ and is captured in the network model as a protein–protein interaction between TPST1 (tyrosylprotein sulfotransferase-1) and the chemokine receptor CCR2. One of the most striking observations in the downregulated glycogene subsets was the decreased expression of enzymes such as DSE (dermatan sulfate epimerase), CHST12 (carbohydrate sulfotransferase-12) and UST (uronyl 2-sulfotransferase) involved in chondroitin/dermatan sulfate biosynthesis, the two immunomodulatory glycosaminoglycans^90–94^.

Significant gap in enrichment analysis databases were identified for a number of glycogenes in the blood sample DEGs. These glycogenes, which are reported to be critical regulators of inflammatory responses, involve OGA (*O*-GlcNAcase) and B4GALT5 (β-1,4-galactosyltransferase-5)^95–99^. Other few important glycogenes identified in this category are lectins involved in the plasma protein clearance, immune cell adhesion and anti-coagulant properties such as C-type lectin receptors ASGR1 (asialoglycoprotein receptor-1), DGCR2 (DiGeorge syndrome critical region gene-2) and thrombomodulin (THBD)^100,101^.

### Enrichment analysis of DEGs—brain samples of MDD subjects

Carrier and channel proteins involved in ion coupled cell transport mechanisms, growth factor signaling pathways as well as protein coding genes involved in neurotransmission and synapse formation were some of the top scoring enriched processes identified from the GSEA and the GO biological process analysis. Similarly, glucose, sialic acid, glycosphingolipid and glycosaminoglycan metabolism, glycosylphosphatidylinositol (GPI) anchor and *O*-glycan biosynthesis were the top scoring enriched processes for the glycogene subsets. Few biological processes associated with ion homeostasis, chemokine mediated signaling pathway, cellular response to cytokine stimulus, ubiquitination, RNA metabolic and biosynthetic processes were also identified from these DEGs (Suppl. Table 15).

**Figure 3:**
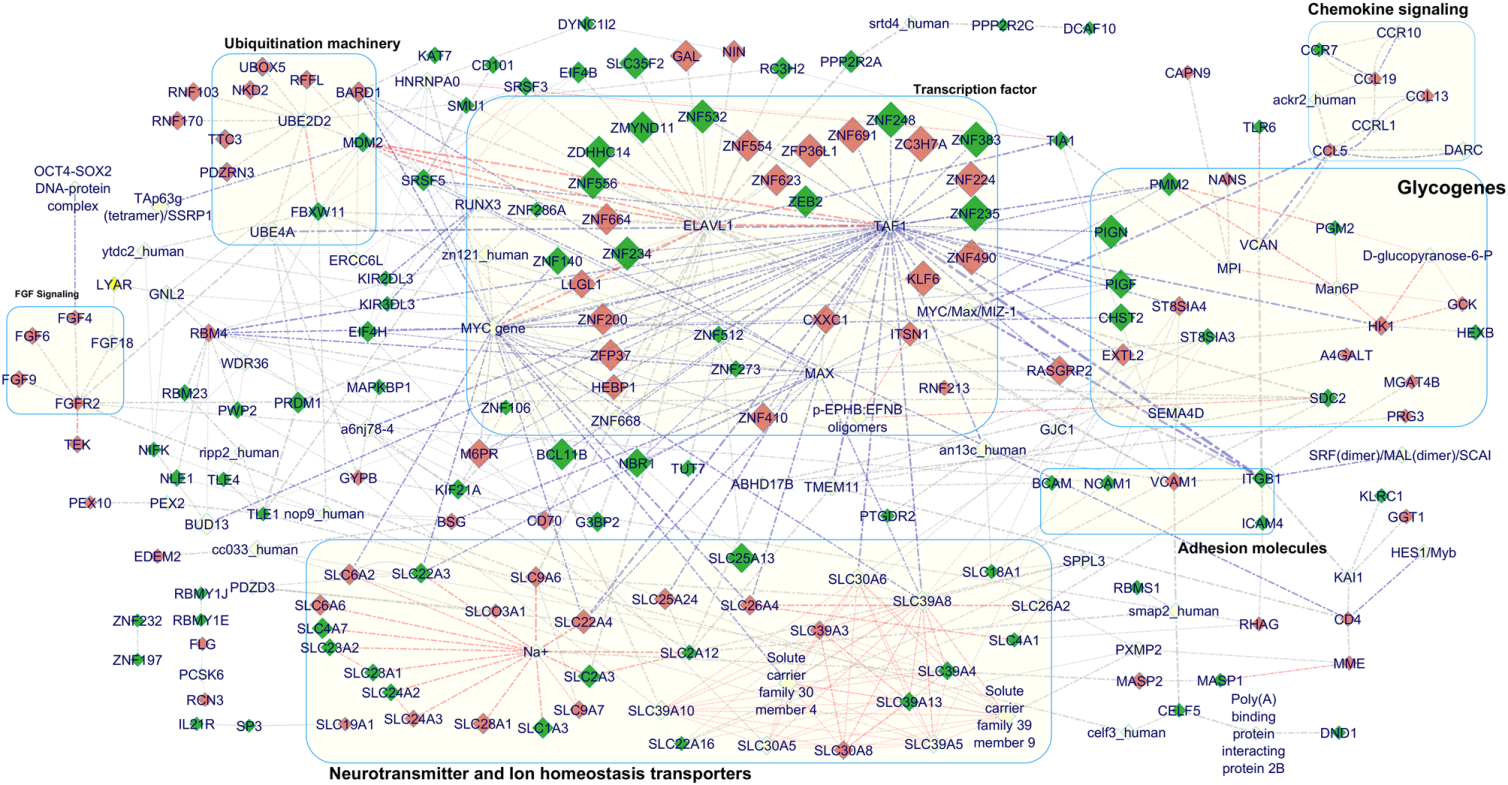
Integrated network model generated for both upregulated and downregulated genes identified from the brain samples of MDD patients using the CPDB induced network modules. The current network model comprises of 218 nodes out of the 243 input genes from the top ranking gene family clusters along with the glycogenes. The compact subnetwork is created by applying a z-score threshold of 20 in the CPDB user interface. Exported network model from the CPDB is manually aligned in the GeneMANIA Force Directed Layout using the Cytoscape visualization tool. Colours of the nodes are adjusted based on gene expression data wherein wine red colour represents upregulated genes and dark green represents the downregulated genes. The DEGs belonging to network modules with maximum representation is highlighted in the graph using light yellow background.

A unique list of 243 DEGs were selected for the induced network model based analysis which extracted a highly interconnected transcriptional network with the ubiquitination machinery, neurotransmitter and ion homeostasis transporters as well as adhesion molecules (Fig. 3). It may be interesting to evaluate whether the altered transcriptional signatures and its regulatory interaction network with the transporter machinery might explain the decreased norepinephrine, GABA and increased levels of glutamate neurotransmitters observed in MDD subjects^102–105^. Moreover, DEGs representing the Fibroblast growth factor (FGF) signaling pathway, dysregulation of which was associated with the anxiolytic and antidepressant function with severely depressed humans^106^, were found to be connected with the ubiquitination and the solute carrier family proteins in the network. Similarly, the upregulated expression pattern of neuropeptide GAL (galanin) among the DEGs was consistent with a previous report^107^ and might indicate dysregulation in pathways involved in mood regulation.

Focused analysis of the glycogene DEGs enabled us to identify few of them to be associated with the transcriptional hub machinery (Fig. 3). These genes are primarily involved in the complex *N*-glycan synthesis, heparan sulfate biosynthesis, ganglioside metabolism, GPI-anchor biosynthesis processes and polysialic acid modification of neuronal cell adhesion molecules^108^. The decreased expression of ST8SIA3 (ST8 α-N-acetyl-neuraminide α-2,8-sialyltransferase-3) involved in the synthesis of c-series ganglioside and HEXB (heoxsaminidase) enzyme involved in regulating GM2 ganglioside content, might indicate altered neuronal repair^109^ synaptic plasticity and neurogenesis^110,111^. Interestingly, in the network model, HEXB gene was associated with the hypoxia regulated hexokinase isoform HK2 and glucokinase enzyme GCK, which are critical regulators of glucose metabolism^110^ and hence might indicate a role of adaptive mechanism to support brain energy metabolism. Similarly, downregulated expression pattern of gene transcripts involved in the GPI-anchor biosynthesis pathway: PIGN (phosphatidylinositol glycan anchor biosynthesis class N) and PIGF (phosphatidylinositol glycan anchor biosynthesis class F) might indicate altered cell surface expression of various proteins that play vital role in neuronal differentiation, synapse development as well as axon guidance^110,112–114^.

### Enrichment analysis of DEGs—blood samples of ADD treated MDD subjects

GSEA and GO biological process categorization of DEGs from the blood samples of ADD treated MDD subjects highlighted phagosome formation, endosomal/lysosomal pathways, positive regulation of cell death and immune responses, antigen processing and presentation, as some of the top scoring enriched pathways. While, for the downregulated gene subsets this include spliceosome, histone modification, mRNA processing, organic cyclic compound or cellular nitrogen compound metabolic process and DNA damage. Similar analysis for the glycogene DEGs yielded sialic acid metabolism, *O-*linked glycosylation and glycosaminoglycan metabolism as the major enriched cellular processes (Suppl. Table 16).

**Figure 4:**
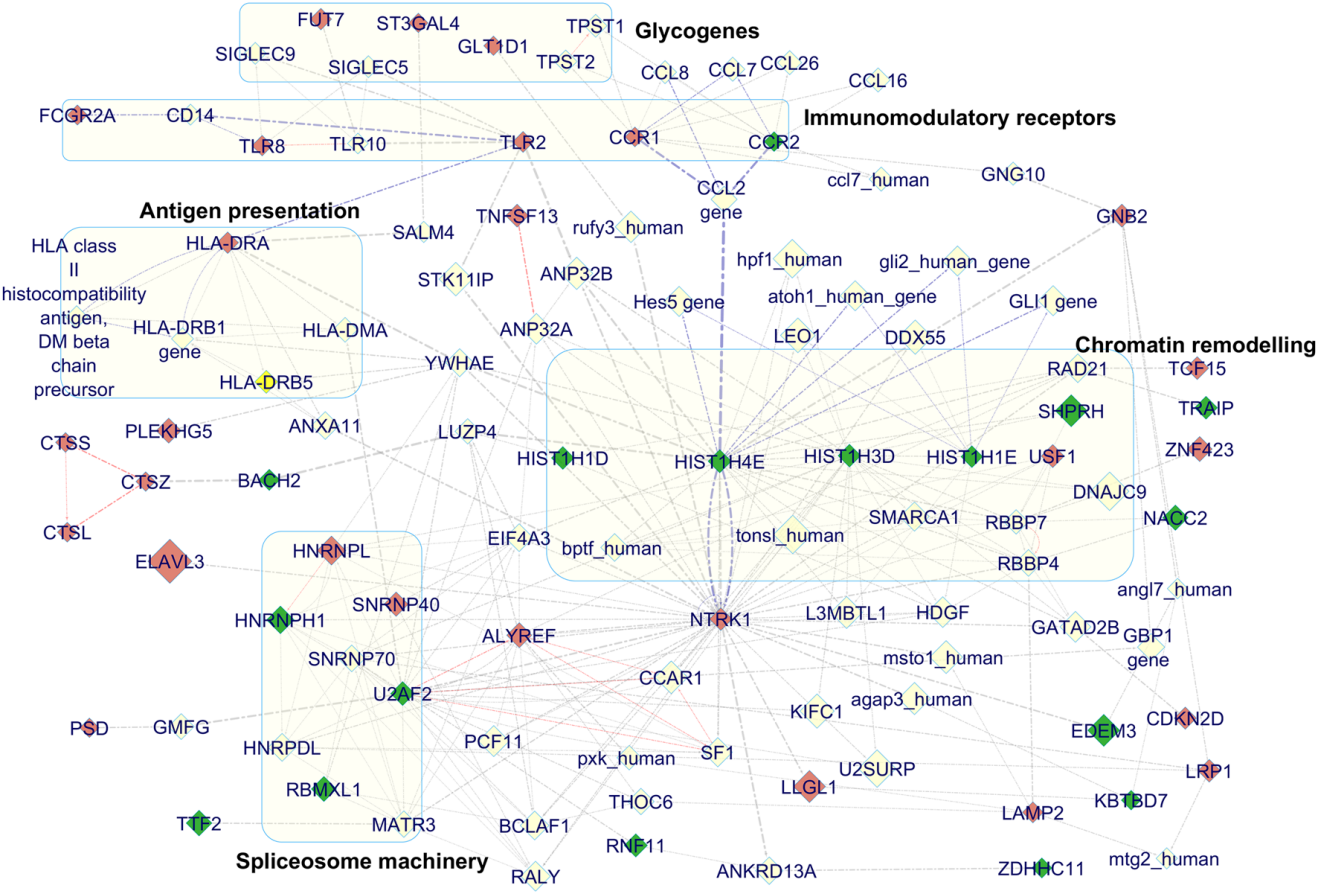
Integrated network model of 82 DEGs from the top ranking gene family clusters identified from the blood samples of anti-depressant drug treated MDD patients using the CPDB induced network modules. The compact network model is created by applying a z-score threshold of 20 in the CPDB user interface. GeneMANIA Force Directed Layout in the Cytoscape visualization tool was used for manually aligning and analyzing the exported network from CPDB. Colours of the nodes are adjusted based on gene expression data wherein wine red colour represents upregulated genes and dark green represents the downregulated genes. The core network modules with maximum DEG representation is highlighted in the graph using light yellow background.

DEGs from the top ranking gene family clusters were subjected to induced network model based analysis by seeding 83 unique gene list integrated from the upregulated and downregulated gene lists, including the glycogenes. Induced network model based analysis using CPDB generated protein–protein interaction networks linking chromatin remodeling and spliceosome machineries with the proteins involved in antigen presentation and immunomodulatory receptor signaling pathways (Fig. 4). Additional literature search on the entities of this seed network suggests a change in the innate immune response pathway^115–119^ as well as co-stimulation of antigen activated lymphocytes^120^ under drug treated condition, which need to be studied in detail. Decreased expression pattern of the linker histones associated with chromatin modification (Fig. 4) suggest that epigenetic modifications are involved in gene expression reprogramming^121^ under drug treated conditions in the peripheral blood mononuclear cells.

Interestingly, among the glycogenes two major glycosyltransferases FUT7 (fucosyltransferase-7) and ST3GAL4 (ST3 beta-galactoside alpha-2,3-sialyltransferase-4) were found to be upregulated and was linked with the TLR and HLD-DRA protein entities (Fig. 4). Another important gene which was found to be upregulated and code for the poly-N-acetyllactosamine synthesizing enzyme, B3GNT8 (UDP-GlcNAc:betaGal-β-1,3-N-acetylglucosaminyltransferase-8), was not found in the induced network model. Due to lack of sufficient information in the induced network model, detailed analysis of the enzymatic reaction and possible glycan structure formation for these enzymes were performed using the GlycoGAIT reaction database. From the reaction table these three enzymes were mapped to 19, 25, 32 and 45 reaction codes (Suppl. Table 12). Manual search for the maximum combination of these reaction codes in the epitope table, lead to the identification of possible glycan epitope structures and were mapped to Lewis^a^- Lewis^x^ (Le^a^-Le^x^), 3′-sialyl Le^a^-Le^x^ (3′sLe^a^-Le^x^), 3′s-Di-Le^a^), 3′s-Di-Le^x^, *O*-Fucose Glycan /EGF Repeat, Sialyl 6-Sulfo LacNAc, Sialyl 6-Sulfo Le^x^, sLe^a^, sLe^x^, sLe^x-i^, VIM-2 (CD65, CDw65, sLe^i-x^) and Myeloglycan structures (Suppl. Table 13). The sialylated Lewis-type blood group antigens as well as the myeloglycan or the polylactosaminolipid structures are known to modulate immune function in both myeloid and lymphoid cells^122–125^.

## Discussion

Diverse integrative network analysis approaches have been adopted in the past to identify the aberrant pathway networks underlying complex diseases by leveraging multiple “-omic” studies^126^. Similar integrative analysis is required to understand the biological implications underlying the phenotypical and physiological expression of complex disorders such as MDD. In addition to the depressive phenotype, MDD subjects are also associated with underlying immune dysregulation, which has been shown to correlate with the metabolic syndrome prevalent in MDD subjects presenting a complex biomarker network^127^.

The undertaking to compile all available data for network and systems biology studies for a single disorder is not a trivial task, limited by analysis and computational techniques currently available, and by the complexity of biological interaction and network. The potential to introduce bias, impacting observations and reported findings exists across all integrated and systems biology networks being subjected to numerous independent datasets, subject to different classification and as is the current case with MDD small-sample datasets. Limitations suffered in common across such studies include, (i) restrictions on the basis of inclusion through the use of keywords and terms; (ii) limited datasets and their size and classification; (iii) sampling and confounding factors; (iv) limiting analysis to top ranked findings, potential missing key molecular interactions as network noise, analysis threshold; (v) subject to level of detail and biological functions assigned in databases.

A number of knowledge databases focusing on MDD as well as molecular meta-analysis data are currently available^26,27,128–131^. There are however, several challenges in the integration of omics data within a network. These extended to but not limited to include experimental and inherent biological noise, differences among experimental platforms, detection bias, and unclear molecular cascades and mechanisms. A common trend, reporting the significant gap in correlation of both genetic and non-genetic biomarkers with the MDD phenotype also exists^132,133^. Commencing this study, a recent umbrella review was selected to give an evidence-based correlation of both proteins and metabolite markers for MDD^56^. Functional enrichment analysis of the MDD associated peripheral markers from this study revealed enrichment of cellular processes associated with inflammatory response, endothelial dysfunction and neurological functions suggesting a close interrelationship between these biological events in MDD condition, which might drive these patients to higher risk of depressive symptoms and metabolic syndrome^134,135^.

From the blood samples of MDD subjects’, gene expression patterns indicated heightened inflammatory signaling pathways and decreased myeloid and lymphoid maturation as well as anti-inflammatory pathways. Results summarized in this study are consistent with the recent findings of increased neutrophil to lymphocyte ratio, platelet volume and activity in MDD subjects^5,77,136,137^. Further to, downregulated gene transcripts identified from the blood samples mapped to complement—coagulation cascades indirectly indicating reduced anti-coagulant activity in MDD subjects. A recent proteomic investigation of plasma based biomarkers in MDD subjects also reported increased expression of proteins involved in coagulation cascades^138^ which might lead to prothrombotic platelet phenotype as demonstrated in a study by Lopez-Vilchez et al.^13^. These findings coincide with the inflammation theory of depression, which suggests a neuro-inflammatory circuit that underlies the development of depression^139–141^.

Gene expression pattern from the brain samples revealed dysregulated neuroprotective mechanisms. The altered gene expression markers of mRNA splicing, translational machinery and ubiquitination machinery, from the brain samples, might indicate an enhanced cellular stress level and altered gene transcription reprogramming. Current analysis, revealed several gene expression markers that correlate with altered levels of neurotransmitters and dysregulated neuronal ion homeostasis which might indicate defective neuron myelination and synaptic signaling^10,103–106,142^ in MDD subjects. Moreover, the genetic findings from a recent GWAS indirectly supports our methodology as the results obtained here in the current analysis were majorly associated with cytokine and immune responses in addition to neuronal development and morphogenesis, showing strong parallel with the GWAS^59^.

In order to understand the impact on gene expression patterns by ADD treatment, relevant gene expression data were queried from the public databases. In this regard, one of the major limitations is in the lack of availability of sufficient brain sample data from ADD treated MDD subjects. DEGs identified from the blood samples of different ADD treated MDD subjects highlight primarily an increase in innate immune response pathway with co-stimulation of antigen-activated lymphocytes. Whether these pathways drive specifically the anti-inflammatory signaling or augment the innate immune response is not clear but this possibility has been proposed and it needs more detailed research to support this hypothesis. A recent systematic review which studied the impact of ADD on innate and adaptive immunity by Bournazos et al., reported high heterogeneity in results and small number of comparable studies^143^. Considering the limited availability of drug treated samples, studies that are more detailed are required to delineate the molecular mechanisms underlying the impact of ADD on immune response pathways, which is considered as one of the factors that underlie treatment-resistant depression in MDD subjects^144^.

Within the GSEA and induced network analysis, DEGs related to glycogenes were analysed. Glycosylation PTM as well as glycoconjugate structures are known to play crucial role in modulating central and peripheral nervous system functions and immune responses^37,145,146^. Results obtained from this study revealed significant gap in databases that support GSEA for glycosylation process related reaction networks. With the aid of GlycoGAIT database and manual literature based analysis, meaningful interpretation for the altered glycogene expression was attempted. From the MDD blood samples transcriptional reprogramming of glycogenes that favor pro-inflammatory response in lymphocytes and monocytes and downregulation of glycosaminoglycans, which have anti-inflammatory and anti-coagulant properties, were identified. The gene expression pattern from the brain samples indicate increased expression of glycogenes involved in synthesis of sialylated structures which are a prominent feature of brain glycoproteins such as those observed on cell adhesion molecules, ion channels and many ligand-activated receptors in neuronal and glial cells^38,147^. Decreased expression of enzymes involved in GPI pathway and in regulation of GM2 ganglioside content might indicate altered brain glycolipid structures, which are crucial for synaptic plasticity and neurogenesis^110,111^. One of the most interesting aspects of the glycogene expression data analysis was the prediction of sialylated Lewis-type blood group antigens as well as the myeloglycan or the polylactosaminolipid structure formation in blood monocytes and leukocytes under ADD treatment condition. Hence, it will be interesting to validate how these specific glycan structures fits with the anti-inflammatory claim of these drugs^148–151^ which can potentially developed as diagnostic markers.

Despite significant advancement in the field of ‘omics’ data analysis and the increasing accessibility to open repositories, no central guideline or platform for an integrative analysis framework has yet been proposed for standardization. For the continued development and acceptance of validity of integrative analysis, standardization at multiple levels is essential. Unlike the analysis of a single dataset, the power of integrative analysis framework stems from the fact that multiple independent data sources can be analyzed to explore trends, similarities or variations in the behavioral patterns of molecular functions and biological processes, which can be correlated with a disease phenotype, outcome and treatment.

The methodology presented here provides an exemplary framework to systematically integrate diverse ‘omics’ data in an open, inclusive and unbiased manner. Researchers, without the need for advanced computational demands can implement and apply the current approach to available datasets requiring minimal computational resources. Presenting a methodological approach, which could be utilized and adapted for exploiting existing datasets without requiring field expertise using publically open and available resources and repositories. Presenting the inclusion of integrative analysis to become a more routine undertaking and building a mass of analysis which can be refined for standardization and proposing validation albeit through replicated integrative studies within single conditions. Using such an approach the study provides novel insights into the molecular mechanisms underlying altered immune response as well as dysregulated neuronal survival maintenance and synaptic functioning in MDD subjects. Furthermore, for the first time altered glyco-genome expression profile from the blood and brain samples of MDD subjects were explored using GlycoGAIT database which might explain the relevance of glycoconjugate structures in modulating the immune responses and neuronal functions.

## Supplementary information

Supplementary information 1.

Supplementary information 2.

Supplementary information 3.

Supplementary information 4.

Supplementary information 5.

Supplementary information 6.

Supplementary information 7.

Supplementary information 8.

Supplementary information 9.

Supplementary information 10.

Supplementary information 11.

Supplementary information 12.

Supplementary information 13.

Supplementary information 14.

Supplementary information 15.

Supplementary information 16.

Supplementary information 17.

Supplementary information 18.

Supplementary information 19.

## Abbreviations

MDD: Major depressive disorder
ADD: Anti-depressant drugs
DEGs: Differentially expressed genes
GSEA: Gene set enrichment analysis
PTM: Post translational modification
GWAS: Genome-wide association studies
