# Supplementary material for: An integrative network analysis framework for identifying molecular functions in complex disorders examining major depressive disorder as a test case
Source: Sci Rep. 2021 May 6;11:9645. doi: 10.1038/s41598-021-89040-7 (PMC8102631; doi:10.1038/s41598-021-89040-7)

**Suppl.Figure_1:** The biological pathway enrichment analysis results for the metabolite markers associated with MDD phenotype. The prioritized list of metabolite markers were selected from the meta-analysis review by Carvallo AF et al. The graph is generated by submitting the HMDB ID of the metabolites into the enrichment analysis module of the MetaboAnalyst platform.


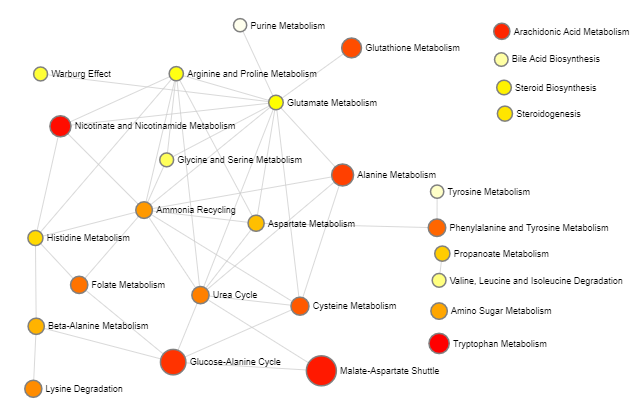

Supplement: Supplementary file 19 — Supplementary information 19. [file 41598_2021_89040_MOESM19_ESM.docx]
